# Exosomes in lung cancer metastasis, diagnosis, and immunologically relevant advances

**DOI:** 10.3389/fimmu.2023.1326667

**Published:** 2023-12-14

**Authors:** Jianhua Zhao, Xiwen Li, Lele Liu, Zhen Zhu, Chunyan He

**Affiliations:** ^1^ Department of Thoracic Surgery, Kunshan Hospital of Chinese Medicine, Affiliated Hospital of Yangzhou University, Kunshan, China; ^2^ Department of Central Laboratory, Kunshan Hospital of Chinese Medicine, Affiliated Hospital of Yangzhou University, Kunshan, China; ^3^ Department of Clinical Laboratory, Kunshan Hospital of Chinese Medicine, Affiliated Hospital of Yangzhou University, Kunshan, China

**Keywords:** exosome, lung cancer, immunotherapy, miRNA, EMT, neovascularization

## Abstract

Lung cancer is a chronic wasting disease with insidious onset and long treatment cycle. Exosomes are specialized extracellular vesicles, at first exosomes were considered as a transporter of cellular metabolic wastes, but recently many studies have identified exosomes which contain a variety of biologically active substances that play a role in the regulation of cellular communication and physiological functions. Exosomes play an important role in the development of lung cancer and can promote metastasis through a variety of mechanisms. However, at the same time, researchers have also discovered that immune cells can also inhibit lung cancer through exosomes. In addition, researchers have discovered that some specific miRNAs in exosomes can be used as markers for early diagnosis of lung cancer. Engineering exosomes may be one of the strategies to enhance the clinical translational application of exosomes in the future, for example, strategies such as modifying exosomes to enhance targeting or utilizing exosomes as carriers for drug delivery have been explored. but more studies are needed to verify the safety and efficacy. This article reviews the latest research on exosomes in the field of lung cancer, from the mechanism of lung cancer development, the functions of immune cell-derived exosomes and tumor-derived exosomes, to the early diagnosis of lung cancer.

## 1 Introduction

Cancer is essentially a disease of uncontrolled growth, unlimited proliferation and metastasis to distant sites, with an insidious onset and a long treatment period (1, 2). The American Cancer Society reported in 2020 that lung cancer currently ranks second in new cancer cases and first in cancer-related deaths worldwide (3, 4). Lung cancer is mainly categorized into two types: non-small cell lung cancer (NSCLC) and small cell lung cancer (SCLC). NSCLC is a predominant type of lung cancer, accounting for approximately 85% to 90% of lung cancer cases. In contrast to SCLC, NSCLC typically exhibits slower growth with better prognosis compared to SCLC (5).

Almost all cell types can secrete extracellular vesicles (EVs). Currently, there is no gold standard for classifying extracellular vesicles (EVs). They can be categorized into microvesicles, exosomes, apoptotic bodies, and more. Different types of EVs exhibit distinct biological characteristics and origins. For instance, microvesicles, ranging in size from 100-1000nm, are directly secreted from the cellular membrane. Apoptotic bodies, generated during cell apoptosis, typically have a size of 50-500nm (6). Various EVs carry different surface markers, allowing for isolation and purification. The biogenesis of exosomes involves the inward budding of the plasma membrane and the formation of intraluminal vesicles (ILVs) within multivesicular bodies (MVBs). Through MVB fusion with the plasma membrane and exocytosis, ILVs are eventually released as exosomes (7).

**Figure 1:**
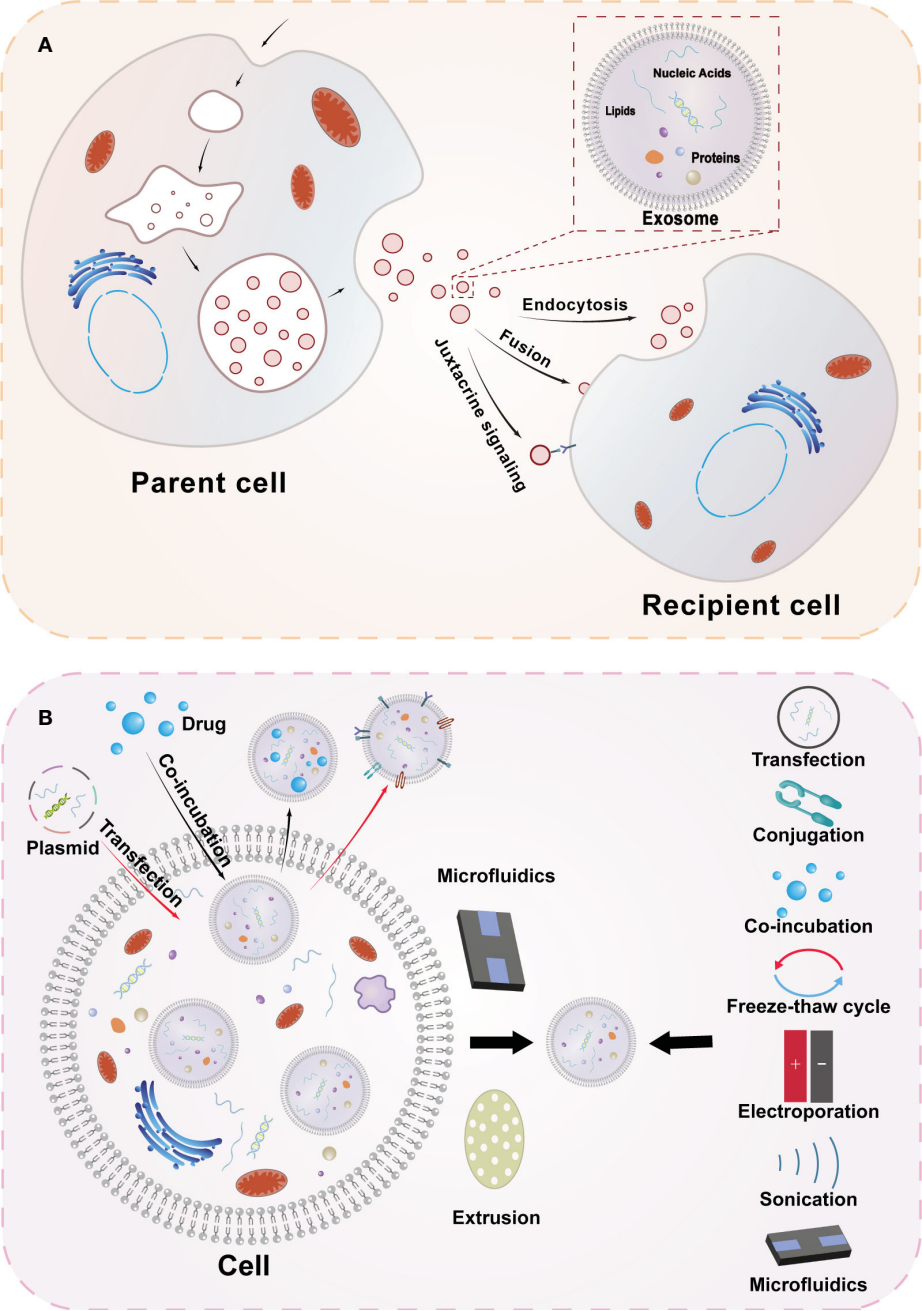
Overview diagram of exosome biogenesis and engineered exosomes. **(A)** Secretion and uptake of exosomes and the composition of exosomes. The cell membrane of the parent cell invaginates to form early endosomes, which subsequently mature into late endosomes and multivesicular vesicles, and the vesicles in the lumen of the multivesicular vesicles are exosomes secreted by the cell after membrane fusion. After exosomes are released to the outside of the cell, they can transmit information to the receptor cells in three ways(Endocytosis, Fusion, Juxtacrine signaling) to achieve the corresponding functions. **(B)** Extraction methods of exosomes and strategies for engineering exosomes. Engineered exosomes can be obtained by modifying exosomes at both cellular and exosome levels. At the cellular level, engineered exosomes can be obtained by processing cells through transfection and co-incubation, etc. At the exosome level, after extracting cellular exosomes through microfluidics and other techniques, information is loaded into exosomes using transfection, cojugation, freeze-thaw cycling, electroporation, sonication and other techniques.

Exosomes are specialized extracellular vesicles produced by the endocytosis pathway with a lipid bilayer closed structure, with diameters ranging from 30-150 nm, and are widely distributed in body fluids including urine, saliva, plasma, cerebrospinal fluid, and bile (7, 8). They contain a variety of biologically active substances, including proteins, DNA, microRNA, lipids, etc (**Figure 1**). Researchers have conducted extensive research to verify their biological functions (9). Cell-to-cell communication can be realized through exosomes, which have also been found to be involved in various physiopathological processes and intracellular mechanism regulation (10).

Exosomes were first discovered and named in 1983. But for a long time afterward, exosomes were not thought to be valuable components until 2007 that it was found to have the capability of intercellular communication, and more studies have subsequently found that exosomes have a variety of physiological functions (11). In other areas such as endocrine, cardiovascular, and ophthalmologic diseases, exosomes can serve as biomarkers for clinical diagnosis (12, 13). The function and contents within the exosomes are heterogeneous due to different origins, and even exosomes secreted by the same cell at different time can exhibit different morphologies and functions. In recent years, exosomes have been found to play an important role in tumor metastasis, immune microenvironment formation, and non-programmed tumor cell death (14).

There are various strategies for exosome isolation, including ultracentrifugation, ultrafiltration, size-exclusion chromatography, precipitation, immunoaffinity-based capture, and microfluidic separation (8, 15, 16). All these techniques allows researchers to unveil the mystery of exosomes. Currently, several researches on exosomes in lung cancer have made good progress, revealing the mechanism in lung cancer progression, drug resistance, and metastasis, and emphasizing the significance of exosomes in the early diagnosis (17). With the increasing knowledge of exosomes, researchers have used innovative techniques to generate engineered exosomes as a novel therapeutic strategy for lung cancer (18).

In this paper, we will briefly introduce the relevant mechanism of exosomes in lung cancer development in recent years. Then based on the previous understanding, we analyze the potential of exosomes as diagnostic markers as well as drug delivery carriers. Finally, we discuss the challenges and potential directions for future applications of exosomes in lung cancer research.

## 2 Exosomes promote the development of lung cancer

### 2.1 Exosomes in tumor drug resistance

Drug resistance refers to the development of tolerance in tumor cells to antitumor therapy, which can greatly reduce the effectiveness of antitumor therapy. Tumor drug resistance can be achieved through a variety of mechanisms, such as altered drug kinetics and enhanced drug efflux and metabolism, affecting tumor cell cycle and promoting proliferation and inhibiting apoptosis (19). Existing studies have shown that exosomes derived from different sources of cells in the tumor microenvironment(TME) can affect tumor drug resistance by influencing tumor proliferation and immunity (20), which include immune cells, tumor cells, and tumor-associated fibroblasts.

Exosomes can act as intermediate carriers to deliver drug resistance information from drug-resistant tumor cells to tumor cells that have not acquired resistance For example, researchers found that cisplatin-resistant NSCLS cells induced by hypoxic environment could secrete PKM2 exosomes to transfer resistance capability to cisplatin-sensitive NSCLS (21). As a result, exosomes can also serve as a drug-carrying tool to inhibit tumor drug resistance in lung (22).

### 2.2 Exosomes in tumor epithelial mesenchymal transition

Tumor metastasis is a major cause of poor prognosis and death, and epithelial mesenchymal transition (EMT) is one of the important pathways for tumor progression and metastasis (23). Normally, EMT helps cells to migrate in the embryo, and tumor cells that undergo EMT show changes of weakened intercellular adhesion and enhanced cell motility, which increases the likelihood of metastasis of tumor cells (24).

Exosomes from tumor-associated fibroblasts (CAFs) enhance cell stemness and EMT in colorectal cancer, thereby promoting tumor metastasis (25). KRT6B in tumor-derived exosomes was also found to promote cancer metastasis by inducing EMT in bladder cancer (26). Similar phenomena were found in kidney cancer (27), liver cancer (28), and breast cancer (29). In lung cancer, exosomes constituting the TME can also mediate EMT in lung cancer, thus promoting metastasis. Researchers found that miRNAs contained in exosomes produced by mysenchymal stem cells under hypoxic environments could elevate the expression of markers associated with EMT in lung cancer by regulating the STAT3 signaling pathway (30). Exosomes from hypoxic LUAD cells could significantly increase the migration of normoxic lung adenocarcinoma cells via SATB2to activate MEK/ERK pathway-mediated EMT (31).

Apart from a direct effect on EMT, lung adenocarcinoma cells may indirectly regulate EMT in lung adenocarcinoma by interfering with exosome secretion from CAFs as well (32). Additionally, different states of tumor cells and other cells in the TME can exert an influence on the EMT process. Exosomes, as one of the constitutive components of the TME, thereby intervene in the progression of the tumor process greatly.

### 2.3 Tumor exosomes and neovascularization

Neovascularization is required for lung cancer growth and metastasis, and is also a factor that affects the prognosis of lung cancer (33–35). The significance of anti-vascularization in lung cancer treatment has been noted early on, and anti-vascular endothelial growth factor (VEGF) drugs have been approved for use in the clinic (36). Studies on the mechanism of neovascularization in lung cancer are more limited, but some specific miRNAs, sirtuin1, and notch pathway have been found to play a role in neovascularization (37, 38). The Tumor Microenvironment (TME) refers to the collective assembly of cells, molecules, and physical factors surrounding a tumor, interacting with tumor cells and influencing the growth, spread, and therapeutic responses of the tumor. Hypoxic conditions are common in the TME. One study indicated that miR-23a in exosomes of lung cancer cells under hypoxia targets ZO-1 protein and prolyl hydroxylase, thereby enhancing angiogenesis and vascular permeability (39), while another study found that miR-197-3p in exosomes of lung adenocarcinoma origin targets TIMP2, TIMP3 to promote neoangiogenesis (40). MiR-3157-3p in exosomes from NSCLS was also found to down-regulate the expression of TIMP2, KLF2, ZO-1, and Occludin, and up-regulate the expression of VEGF, MMP2, and MMP9, ultimately leading to increased angiogenesis (41). Currently, anti-angiogenic drugs, such as bevacizumab, apatinib, abciximab, have been applied in the clinical treatment of lung cancer patients, with good efficacy. Since lung cancer exosomes is believed to be one of the mechanisms of neovascularization, and existing research found that the miRNAs therein seem to be the key, one may opens up the idea that if the application of the inhibitors of these miRNAs, or the inhibition of secretion of tumor exosomes, is possible to achieve good results? Of course, this requires more in-depth research to verify the safety and efficacy.

### 2.4 Tumor exosomes in immune cells regulation and immune efficacy of related research

The human immune system recognizes and removes tumor cells from the body, and tumor cells can evade immune recognition and removal by inhibiting immune cell proliferation or activation. For example, tumor-derived exosomes have been found to alter mitochondrial function to inhibit the proliferation of cytotoxic T cells (42). Natural killer cells(NKC) are sentinel cells of the immune system (43) that recognize tumor cells and remove them without additional activation. During immune evasion of tumor cells, a large number of bioactive molecules contained in tumor cell-derived exosomes, such as transforming growth factor-β (TGF-β), programmed death ligand (PD-L1), ligand for natural killer cell activated receptor NKG2D (MICA/B), apoptosis-associated protein Fas-L, etc., will recognize the cognate receptor on NK cells and abolish the anti-tumor activity. Regarding macrophages, tumor-derived exosomes have also been found to inhibit polarization, achieving immunosuppression and allowing tumor progression (44, 45).

The lymphatic system is responsible for immune cell activation, and the researchers found that exosomes secreted by tumor cells containing immunosuppressive protein PD-L1 can inactivate immune cells to protect themselves from being killed (46). This observation gives clue to researchers that if one can inhibit some tumor cell-derived exosomes, it will also be possible to address the dilemma of insensitivity to immune checkpoint inhibitors in many patients. However, this also raises new challenges in developing medications that can act on tumor exosomes.

Besides the effect of tumor-derived exosomesaffect on immune cells, exosomes secreted by immune cells also exert impact on the tumor immune response. In addition to recognizing and killing tumor cells, NK cells exosomes also modulate the immune response of T cells (47) and are cytotoxic to cancer cells (48). Exosomes secreted by M1-polarized macrophages were found to promote M1 polarization of macrophages, thereby enhancing anti-tumor immunity and inhibiting tumor growth (49).

### 2.5 Research progress of exosomes in the early diagnosis of lung cancer

Large number of researches have validated the function of exosomes in the progression of lung cancer (50), and people are also thinking about the role of exosomes in the clinical context. Early diagnosis of lung cancer can significantly improve the survival rate of patients (51, 52). Although low-dose computed tomography (LDCT) has improved the diagnosis rate of lung nodules, LDCT has poor specificity and is prone to unnecessary surgery, so non-invasive means to diagnose benign and malignant lung nodules is a very promising research direction.

**Figure 2:**
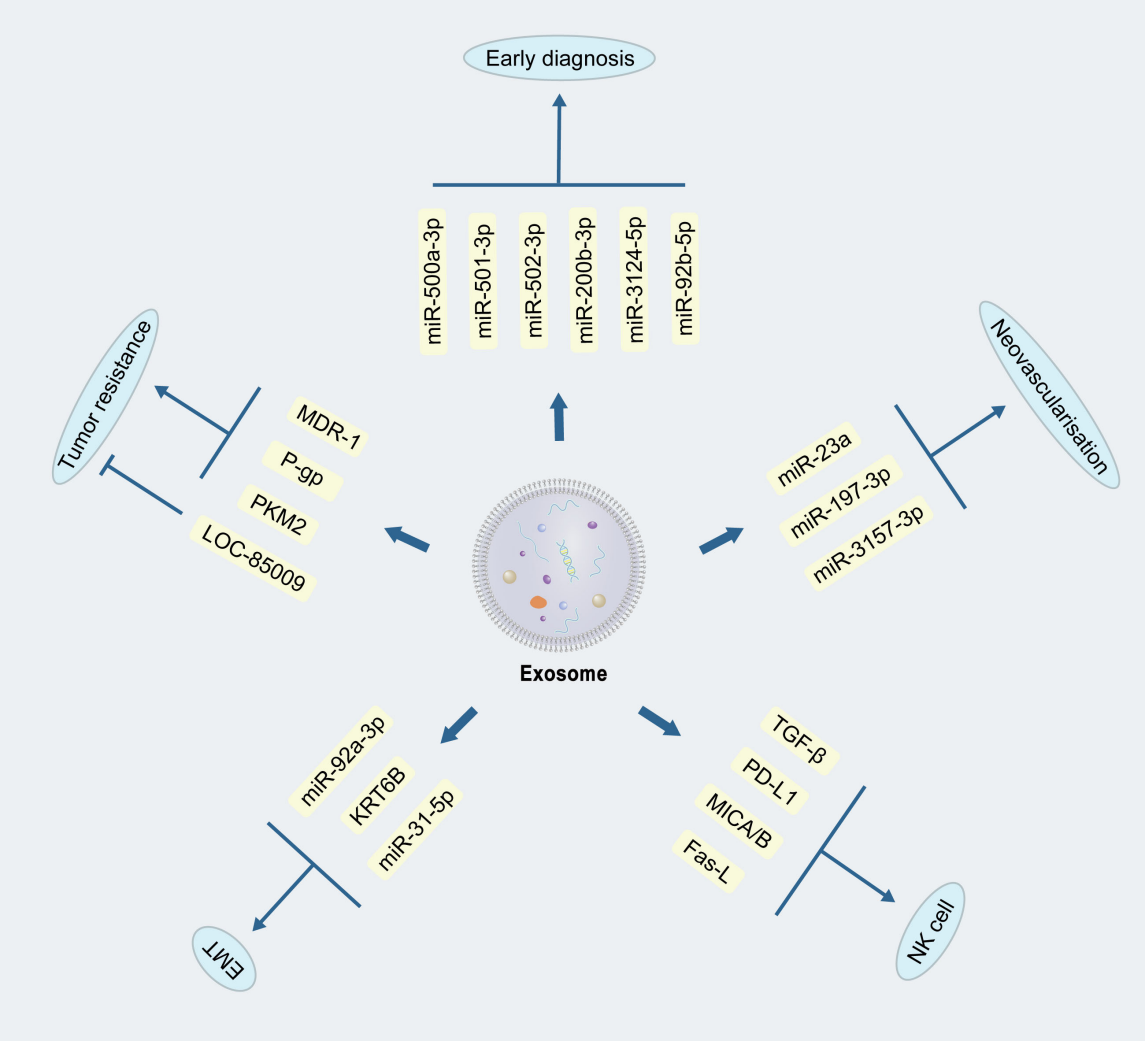
Mechanisms of exosomes in the development of lung cancer. Exosomes can promote or inhibit lung cancer development through different bioactive substances, as reflected in tumour drug resistance, epithelial-mesenchymal cell transformation, neovascularization, immunomodulation, and early diagnosis.

Based on the changes of miRNA profiles in plasma exosomes, researchers identified miR-500a-3p, miR-501-3p, and miR-502-3p, up-regulated within lung cancer, which revealed the possibility of early diagnosis of lung cancer by plasma exosomes (53). Subsequently, another researcher team constructed a diagnostic model to distinguish benign and malignant lung nodules based on plasma exosomal miRNAs, which was also reported encouraging outcomes for lung nodules with a diameter of less than 1 cm (54). Combined with the current deep learning algorithm Shin et al. validated the feasibility of human plasma exosomes as potential tumor-associated biomarkers (55). Subsequently, a set of miRNAs, i.e., miR-200b-3p, miR-3124-5p and miR-92b-5p, in serum exosomes could be used as diagnostic and prognostic markers for SCLC (56) (**Figure 2**). Another study showed that exosomes of SCLC cells with molecules such as Hippo, Rap1, and Wnt could also be used as indicators to determine prognosis (57). At present, a series of studies have verified the feasibility of exosomes in the early diagnosis of lung cancer. Combining the advantages of non-invasiveness of exosomal examination with existing tests is expected to enhance the early diagnosis and prognosis of lung cancer.

## 3 Discussion

Exosomes act as a carrier of information passed from cell to cell, altering the responsiveness of the immune system and the microenvironment. Understanding how the immune system reads the information in exosomes and how exosomes affect immune cells is important for the development of new strategies that will allow immune cells to behave effectively. For example, exosomes secreted by lung cancer cells contain miR-21 and miR-29a, which can be taken up by surrounding tumor-associated macrophages (TAMs) to influence cytokine secretion (58), which regulate tumor growth and metastasis. Exosome secretion has been found to be pH-sensitive, with the pH of the tumor microenvironment affecting both exosome secretion and the contents (59). The acidic environment in the tumor microenvironment tends to lead to the death of immune cells. This is why some researches have proposed that neutralizing the acidic environment of the TME using proton pump inhibitors or other buffering therapies can reduce the immune escape of tumor (60), and these may be the directions in the future to produce an anti-tumor therapy by restricting the function of tumor exosomes. Exosomes may be a potential treatment for tumors as well. While drug therapy can lead to drug resistance and cell therapy can lead to the risk of so-called “cytokine storm” in the body, exosomes may be able to circumvent these side effects. In addition, exosomes have been shown to cross the blood-brain and blood-testis barriers, a vital property in the context of cancer cells, since these cells can evade elimination by immune cells through various immune escape mechanisms. Nevertheless, exosomes have the potential to bypass such mechanisms and still target and kill cancer cells effectively.

One of the problems in the development of exosome-related tools is that the content is variable and complex. On the other hand, the development of engineered exosomes allows researchers to use exosomes as carriers, in which specific content is loaded. This technology may be one of the future drug delivery strategies for targeting tumor cells, and good results have been detected in lab. Still, more research is needed.

Apart from the deepened understanding of exosomes in lung cancer in recent years, there is still much limitation in the clinical application of exosome-related knowledge and techniques. First, the difference in the delivery mechanisms of exosomes from various cancer cells as compared to normal cells are not clearly investigated. Unveiling the factors influencing category and amount of cargo within the exosomes will remarkably help us gain a better picture of cancer biology. Secondly, one should keep in mind that the distribution and concentration of exosomes is not even across different systems. For example, blood-brain barrier has a significant impact on exosome biology and induces a divergence between those in circulation and in cerebrospinal fluids (61, 62). Does alveolar-capillary barrier has similar effect should be depicted in the future, as this will provide valuable clue to the development of better laboratory methods with more accuracy. Third, a dynamic observation of exosomes from sputum, blood, and within the tumor in response to therapies is still poorly conducted in detail. A comparison and integration of these information can shed light on cancer biology and therapeutic targets.

People first discovered exosomes in the culture fluid of reticulocytes, which was then thought to be nothing more than a garbage truck for cells to remove waste. Decades later, exosomes once again attract much attention with a new face. With the deepening of research, it is found that exosomes have rich biological functions, which can regulate the growth of the cells, signaling. Then, researchers found that exosomes have specificity, and some of their special components can be used as markers for identifying exosomes. (fig4, Progress in the understanding of exosome research, three stages). Researches also found that the contents in exosomes are related to the cell state and cell origin, which also laid the foundation for exosome-based therapeutics and diagnostics. In recent years, the proposal of engineered exosomes has expanded the applications of exosomes, allowing researchers to modify the surface of exosomes to make them targeted or to evade recognition by other cells. The contents of exosomes can also be customized to carry specific drugs or miRNAs. In short, with the deepening of research, the functional development of exosomes will be more complete.

## 4 Limitation

Apart from the deepened understanding of exosomes in lung cancer in recent years, there is still much limitation in the clinical application of exosome-related knowledge and techniques. First, the difference in the delivery mechanisms of exosomes from various cancer cells as compared to normal cells are not clearly investigated. Unveiling the factors influencing category and amount of cargo within the exosomes will remarkably help us gain a better picture of cancer biology. Secondly, one should keep in mind that the distribution and concentration of exosomes is not even across different systems. For example, blood-brain barrier has a significant impact on exosome biology and induces a divergence between those in circulation and in cerebrospinal fluids (61, 62). Does alveolar-capillary barrier has similar effect should be depicted in the future, as this will provide valuable clue to the development of better laboratory methods with more accuracy. Third, a dynamic observation of exosomes from sputum, blood, and within the tumor in response to therapies is still poorly conducted in detail. A comparison and integration of these information can shed light on cancer biology and therapeutic targets.

## 5 Conclusion

Exosomes of lung cancer origin can influence the metastasis and development of lung cancer through multiple mechanisms, while exosomes secreted by immune cells can also influence the progression of lung cancer. Exosomes have the potential to be a complementary means of early diagnostic tool for lung cancer, but more exploration is still needed.

## Author contributions

JZ: Writing – original draft, Conceptualization, Methodology. XL: Writing – original draft, Writing – review & editing, Data curation. LL: Writing – original draft, Data curation. ZZ: Writing – review & editing, Methodology. CH: Writing – review & editing.
